# Wolf Rock lighthouse: past developments and future survivability under wave loading

**DOI:** 10.1098/rsta.2019.0027

**Published:** 2019-08-19

**Authors:** A. C. Raby, A. Antonini, A. Pappas, D. T. Dassanayake, J. M. W. Brownjohn, D. D'Ayala

**Affiliations:** 1 School of Engineering, University of Plymouth, Drake Circus, Plymouth PL4 8AA, UK; 2 Department of Hydraulics Engineering, Delft University of Technology, 2628 CN Delft, The Netherlands; 3 Civil, Environmental and Geomatic Engineering, UCL, Gower Street, London WC1E 6BT, UK; 4 College of Engineering, Mathematics and Physical Sciences, University of Exeter, Exeter EX4 4QF, UK

**Keywords:** lighthouses, wave loading, modal testing, wave statistics, structural response

## Abstract

Lighthouses situated on exposed rocky outcrops warn mariners of the dangers that lurk beneath the waves. They were first constructed when approaches to wave loading and structural response were relatively unsophisticated, essentially learning from previous failures. Here, we chart the evolution of lighthouses on the Wolf Rock, situated between Land's End and the Isles of Scilly in the UK. The first empirical approaches are described, followed by design aspects of the present tower, informed by innovations developed on other rocky outcrops. We focus on a particular development associated with the automation of lighthouses: the helideck platform. The design concept is described and the structure then scrutinized for future survivability, using the latest structural modelling techniques of the entire lighthouse and helideck. Model validation data were obtained through a complex logistical field operation and experimental modal analysis. Extreme wave loading for the model required the identification of the 250-year return period wave using a Bayesian method with informative prior distributions, for two different scenarios (2017 and 2067). The structural models predict responses of the helideck to wave loading which is characterized by differential displacements of 0.093 m (2017) and 0.115 m (2067) with associated high tension forces and plastic strain.

This article is part of the theme issue ‘Environmental loading of heritage structures’.

## 1. Introduction

Eight miles off the most southwesterly point of the UK, halfway between Land's End and the Isles of Scilly, lies the Wolf Rock lighthouse. Only the lighthouse itself is visible at high tide, with no sign of the treacherous rock that it marks. The surface-piercing rock is steep-sided and surrounded by water depths of around 60 m. From a wave transformation point of view, this means that waves have the potential to reach the tower with little of their energy being dissipated by breaking onto the rocks. The lighthouse, therefore, has the potential to experience the largest wave impacts of any of the 20 rock lighthouses around the UK. These lighthouses are still essential forms of navigation, even in the era of GPS, as the UK and Irish General Lighthouse Authorities (GLAs) do not regard such satellite systems as failsafe.

**Figure 1.**
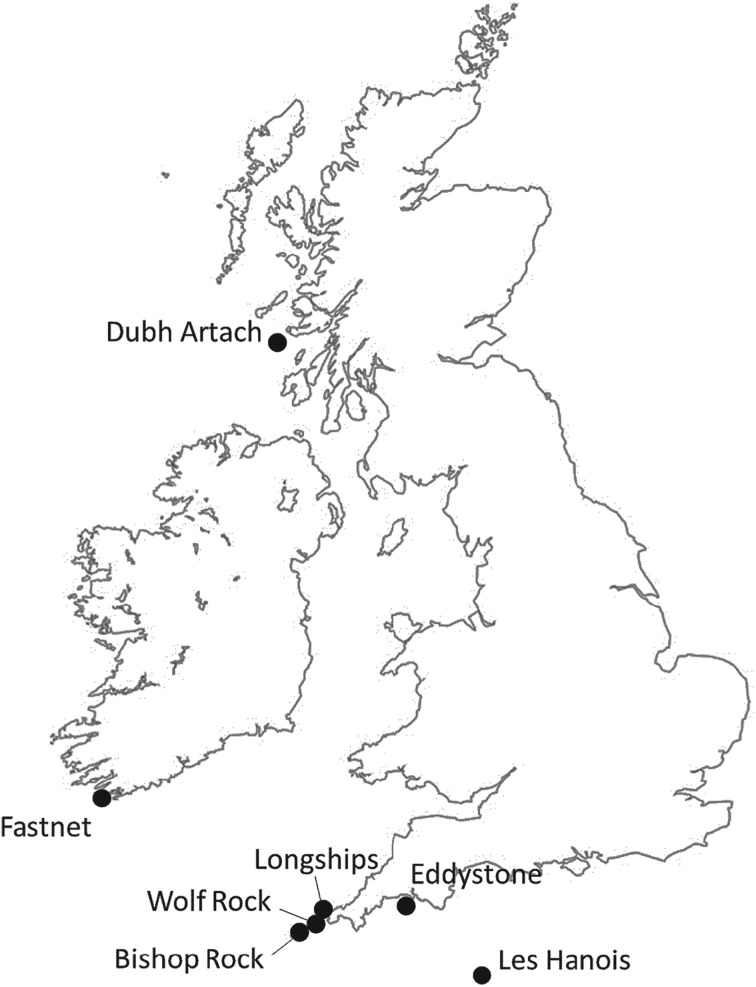
Map of the British Isles indicating the locations of the seven lighthouses investigated in the STORMLAMP project.

For many years, lighthouses were permanently manned by lighthouse keepers, though in recent times they have become fully automated. Still, engineering crews stay for up to two weeks at a time, depending on maintenance requirements. Some of the anecdotal accounts from previous lighthouse keepers stationed on Wolf Rock are recorded in the Editorial of this Special Issue. While the accounts are fairly alarming in nature, none of the structural vibrations arising from wave impacts have until now been quantified, except at the less-exposed Eddystone where maximum displacements were modest [1]. However, the towers of Wolf Rock, Longships, Bishop Rock, Les Hanois, Dubh Artach and Fastnet (figure 1) face much worse wave climates and so have been investigated in the STORMLAMP project, funded by the UK Engineering and Physical Sciences Research Council. The UK and Irish GLAs need to know the conditions of these assets and whether they will remain operational into the future in the light of climate change, with the increase in sea level and the possibility of increased storminess. Lessons learned from this project will be applicable to other rock lighthouses internationally. Also, methods of monitoring remote structures are relevant for other situations, and the hydrodynamic and structural modelling of wave impacts on the lighthouses has relevance for a host of coastal and offshore structures.

This paper presents the adaptation of the earliest beacons and the lighthouse on the Wolf Rock, over a period of nearly 200 years. It particularly charts the latest chapter of its story, with results from ongoing monitoring and modelling that shed light on the current and future stability of the lighthouse. The paper is organized as follows: §2 presents the chronological developments of structures on the rock over the past two centuries; §3 describes the field modal testing and experimental modal analysis of the current lighthouse, providing valuable information on the structural behaviour to validate the structural models; §4 presents the Bayesian method to predict wave loading that will be applied to the structural model for current and future scenarios; §5 provides details of the structural model with particular focus on the helideck behaviour under the wave loading; and §6 concludes with a discussion.

## 2. Engineering developments at Wolf Rock

### (a) Initial beacons

**Figure 2.**
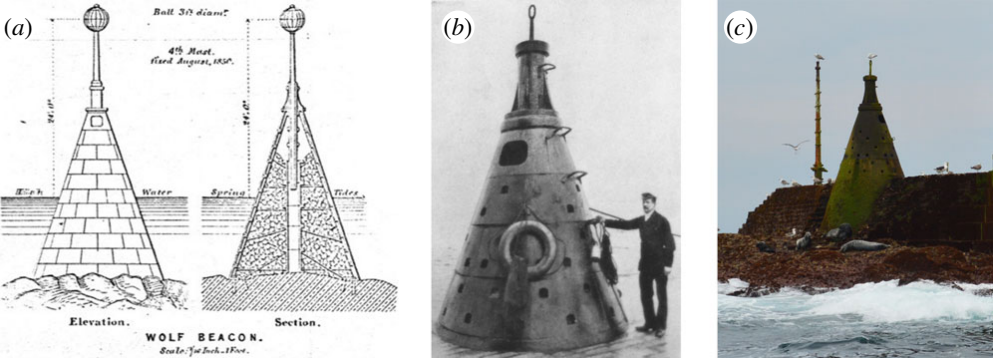
Wolf Rock beacon: (*a*) archive drawings of the elevation and section views (reproduced by kind permission of Trinity House); (*b*) photograph taken in 1909 by a journalist from the *Pall Mall Magazine* after two unsuccessful attempts at visiting, clearly showing the cup holds that have undoubtedly been used to resist being swept into the sea on multiple occasions, including an event recounted by the helideck designer, who in the early 1970s had to embrace the cone when he was entirely engulfed by a wave for a period of several seconds (S. Simmons, personal communication, 2019) and (*c*) photograph taken of the beacon and wildlife enjoying calm conditions at low tide in 2018 (reproduced by kind permission of Ken Trethewey). (Online version in colour.)

At high tide, the Wolf Rock is completely covered. In the past this presented a severe hazard to mariners; indeed, three Elder Brethren of Trinity House were reported to have drowned close to the spot during a visit [2]. The first solution to marking the hazard was in 1795 when a simple 101 mm diameter pole of wrought iron was fixed into the rock, with a plug of lead, and the support of six wrought iron stays also fixed into the hard, dark felspathic porphyry. However, it was ‘soon’ lost to the sea [3]. The next solution, in 1836, was a 6.71 m high cast iron cone of 3.66 m base diameter which was filled with solid masonry [4]. The cone was topped by a series of four different masts and balls, as each was subsequently washed away. A final design of 0.23 m diameter iron mast and 0.91 m diameter ball lasted 19 years, only being removed when a new structure was erected. The construction cost of the beacon was £11 298 [3]. Figure 2 shows details of the beacon.

### (b) A rock tower

Despite the beacon being painted white and red, it was not visible at night [5], so a new structure was required. This responsibility fell to James Walker, president of the Institution of Civil Engineers between 1834 and 1845 [6], who had already designed and built similar stone towers on Bishop Rock and the Smalls [7]. A century earlier, the Eddystone lighthouse, which was designed by Smeaton, had three crucial details to overcome wave action, which would be incorporated into the designs of Walker and Douglass. These were: a curved concave profile, 16 sector stone blocks for each ring and the dovetailing of the stone blocks fixing the sectors into each other, to render each course monolithic [8]. Wolf Rock was ultimately designed with a variable number of sector stone blocks per ring, ranging from 16 blocks for the lower rings to nine blocks for the upper ones. It should be borne in mind that modern concrete technology was in its infancy at the time and indeed, Smeaton is often credited as the first to use hydraulic lime in the joints of the Eddystone lighthouse. To prevent the relative sliding of successive courses, Smeaton also included nine marble dowels and two trenails in each single block, vertically linking the courses of the solid base. Walker was familiar with this construction having been in charge of its maintenance as Principal Engineer for Trinity House, at the same time as the first design for the Wolf Rock lighthouse was conceived in 1860 [6].

**Figure 3.**
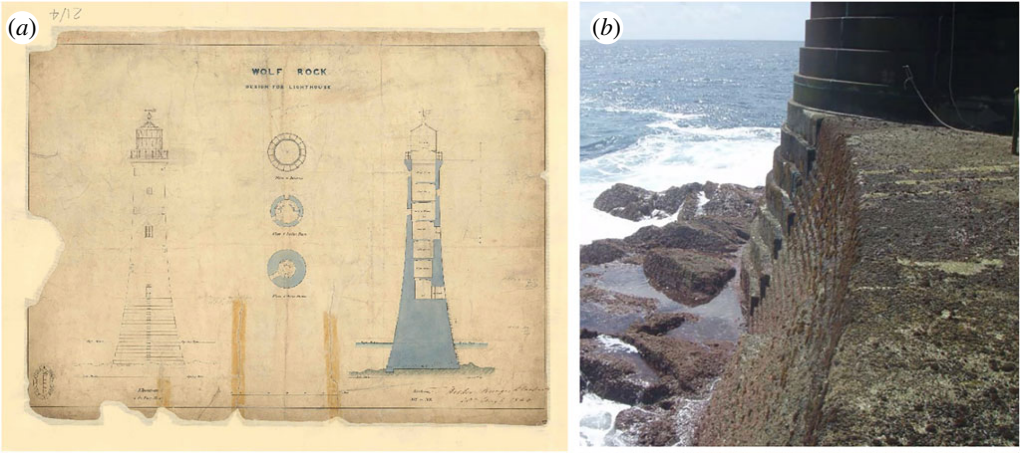
Wolf Rock lighthouse design (*a*) original drawing signed by James Walker (reproduced by kind permission of Trinity House) and (*b*) detail of the stepped lower courses (reproduced by kind permission of Ken Trethewey). (Online version in colour.)

Although plans for a lighthouse had already been put forward by Stevenson in 1823, it was considered too impractical and dangerous to erect anything more than a beacon [9]. The design by Walker was similar to the lighthouse already erected on Bishop Rock completed in 1858, with a slightly wider and taller solid base. The major novelty of the Wolf Rock design is the dovetailing of the top of the stones as well as the sides. This same technique was employed at Les Hanois, also designed by Walker, but completed by William Douglass in 1862. Moreover, 51 mm diameter gun-metal joints were used between courses and to fasten the tower to the rock. With its iconic concave elliptic frustum curve [3] with a maximum diameter of 12.68 m at its base, Wolf Rock lighthouse stood 35.33 m from foundation to gallery, the lowest 11.98 m being solid except for a water tank (figure 3*a*). The lowest 20 courses were of a stepped design, seen more clearly in figure 3*b*, this being an attempt to reduce the wave loading and subsequent runup [5].

James Douglass took over the construction of Wolf Rock, following Walker's sudden death in 1862, and was joined by his brother William as resident engineer. It is not entirely clear whether the vertical dovetailing solution was his or Walker's own. It had the benefit of locking and fastening the stones from one course to the ones above and below, preventing relative rotation and hence further enhancing integrity and robustness of the construction. Douglass was challenged at an Institution of Civil Engineers meeting about the use of dovetailing. He was asked whether recent improvements in the manufacture of Portland cement were considered effective in delivering sufficient tensile strength to a stone conglomerate, to make it act as a monolith in response to the waves action. Douglass responded that the dovetailing and related extra stone added less than 1% to the overall cost and they ensured ‘permanent stability and safety of the whole structure’ [3]. Indeed, there was not sufficient evidence as to the durability of the binding strength of Portland cement and concrete and on its stability in aggressive environments.

Construction progress at Wolf Rock was painfully slow due to the severe constraints of working on such a small rock, which rarely pierced the sea surface. In fact, in the first year of construction, only 83 working hours were completed on the rock. The first part of the structure to be built was a 413 m^3^ landing stage which comprised 15 000 blocks, each 76.2 kg [5]. In 1865, in the later stages of construction, a partial course of blocks that had been placed the previous summer was frustratingly lost, apparently due to being hit by a ship's mast that was cut off during a storm [3]. However, by 1870, the lighthouse was completed, lit and inhabited by its first lighthouse keepers. The entire cost was £62 726 [3].

### (c) Helideck structure

Among all the Trinity House rock towers, the lighthouse keepers on Wolf Rock suffered most from sea conditions affecting their periodic removal from the station; the average overdue time for reliefs was 43 days (S. Simmons, personal communication, 2019). Since the keepers union had negotiated that the tender bringing the relief crew would stay in the vicinity if they were more than 7 days overdue, the Wolf Rock lighthouse tied up these support vessels and the scores of crew they had on board. Financial pressure was brought to bear by both Trinity House's political overseer (the Board of Trade) and its de facto paymaster (the Shipowners Conference) to implement a helideck which would ensure more efficient crew reliefs. Another driver for the design of a helideck was the forthcoming automation of the towers, already being implemented for lighthouses on land. In an era when lighthouse keepers would no longer be on the lighthouse, but there was an operational problem (a ‘casualty’ in Trinity House parlance), it would be necessary to deploy engineers to the tower with minimal delay.

While helidecks had been constructed on oil platforms prior to this time, the innovation of an over-lantern helideck on a lighthouse cannot be exaggerated. The task fell to a relatively junior Trinity House Civil Engineer, Steve Simmons, who had recently entered the company, having previously designed the Inner Dowsing oil platform deck. The design would require numerous visits to the tower, being winched across on a rope from a boat, suspended in a shoulder harness while being dragged through the water and bumped up the side of the tower. The requirement for the Wolf Rock helideck was for a 45-year design life, with a 15-year maintenance period. It was not to cause any instability in the lighthouse nor was it to obscure the lantern. There was scant guidance available, limited to a few recommendations on Offshore Helicopter Platforms (produced by the Offshore Platforms group of the Marine Division of the Board of Trade): to have sufficient clearance beyond the rotors for personnel and stores, to be able to bear the load of the largest helicopters likely to heavily land and to have a safety net. The implementation of a net was to save at least one lighthouse keeper who was hit by an inadequately secured helicopter loading door and knocked off the main helideck (S. Simmons, personal communication, 2019).

**Figure 4.**
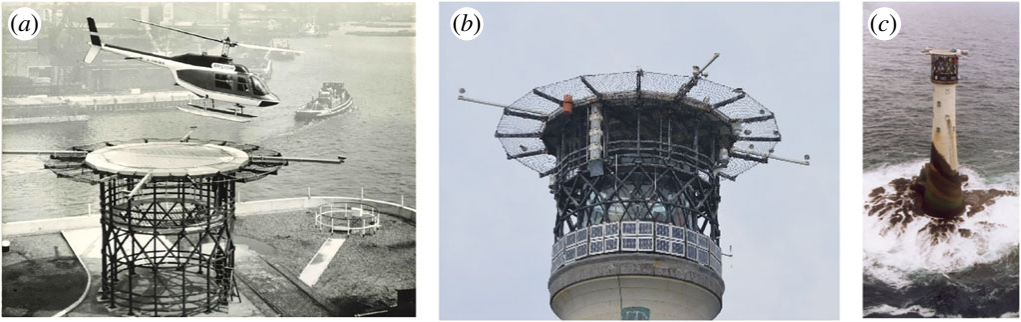
Wolf Rock helideck (*a*) trial at Trinity House's Blackwall depot, (*b*) contemporary view (reproduced by kind permission of Ken Trethewey) and (*c*) the ‘new’ vernacular of a helideck-mounted lighthouse (reproduced by kind permission of Ken Trethewey). (Online version in colour.)

The final design, shown in figure 4, was based around a series of cross-linked portal frames, of box section steel, with cantilever beams providing torsional rigidity. Diagonal raked drums minimized the loss of light out, and provided a visual accent of the lantern window panels. The massive handrails contribute to the rigidity of the structure. The steel was shot-blasted, metal sprayed then treated with polyvinyl butyral sealer. Non-structural parts (walkway, ladders, deck panels and hatches) were fabricated from aluminium. The addition of four cantilevered poles with flat discs on the ends were a necessary addition to ensure a helicopter pilot could judge where they were landing, as the helideck would be completely obscured from within the cockpit.

Wave loading was considered, though the version of the Morison equation [10] used in the early 1970s would not have included dynamic (impulsive) loading. It was anticipated that spray from waves would reach the helideck, so the aluminium plates were held *in situ* with shear pins designed to fail beyond a certain load. In the first year of operation, deck panels were lost twice due to the severity of the conditions, against the expectation that this would happen once every 20 years.

The construction of the helideck was rehearsed at Trinity House's Blackwall depot (figure 4*a*), enabling issues to be overcome on dry land. It also provided the opportunity to assess whether keepers would be willing to climb out of a helicopter onto such a small platform at height; if the keepers had been resistant, then the platform would merely have been used for winching. Actually, the down-draft from the helicopter was sufficient to reduce the effect of disconcerting side gusts on personnel. The construction at sea took place in the summer of 1973 by a team of three men. The first activity was to remove the domed lantern roof and replace it with a much flatter structure to minimize the required height of the helideck support structure. The structure was secured to the lighthouse by foundation bolts that were sunk vertically into the top of the tower at a distance of about 5 m, with an epoxy grout. All elements of the helideck were designed to be handled by a maximum of two people. Materials were transferred laboriously from the MV Strathclyde which needed to make frequent trips back to the base at Penzance, as storage on the tender was limited (S. Simmons, personal communication, 2019; [5]).

The helideck was used for the first time for the relief of lighthouse keepers on 3 November 1973 [11]. Innovations in helicopter design have resulted in a reduction in their weight relative to payload, such that helidecks are still well within their design loads. With the installation of the helideck, ‘only a fog or tearing gale’ would be able to prevent crew relief by helicopter [7]. A Principal Keeper stationed on Wolf Rock over a period of 9.5 years often mentioned (G. Douglass-Sherwood, personal communication, 2019) how he felt the tower was prone to judder more since the helideck was commissioned. Results presented in §3 indicate that the helideck will certainly have affected the dynamic properties. The estimate provided to the Trinity House Light Committee for the construction of the helideck in 1972 was just £15 750.

### (d) Lights, electrification and automation

A series of other modifications have been made on the Wolf Rock lighthouse, albeit requiring less heroic endeavour. The original lamp was a first-order optical apparatus of 2.58 m height and 1.73 m diameter which rotated courtesy of a clockwork mechanism that required winding every 4 h. Electrification of the lighthouse in 1955 resulted in an electrically driven revolving pedestal, manufactured by Chance Brothers, the original company. A fourth-order catadioptric apparatus was also installed and a 1 kW tungsten filament lamp fitted [5]. The arrangement produces one flash every 15 s and can be seen at a distance of 16 nautical miles. The automation of the tower and the associated departure of the lighthouse keepers happened in 1988. Fifteen years later, solar photovoltaic panels were fitted around the support structure of the helideck, providing the latest adornment to the lighthouse (figure 4*b,c*).

## 3. Field modal tests

### (a) Modal test objectives and planning

The modal testing of the STORMLAMP project aimed to provide information on the structural condition, delivered in the form of a comprehensive set of modal properties: the natural frequencies, damping ratios, mode shapes and modal masses. These would be useful for calibrating the structural numerical models of the lighthouses and for estimation of breaking wave impact loads during monitoring.

The choice of test methodology was between ambient vibration testing, requiring only a set of accelerometers and recorder, and forced vibration test, which in addition requires a mechanical shaker and power amplifier. The only information to inform the decision was data from Eddystone Lighthouse [1] which suggested a first mode with natural frequency at 4.4 Hz. Trinity House drawings of the masonry courses, and vibration mode shape assumed to be linear from basement to crown, provided an estimated modal mass of 600 tonnes. Experience with modal testing of other civil structures has shown that such a mass can be readily excited by an APS 113 HF electrodynamic shaker operating within its optimal range producing peak output of approximately 186 N. This was expected to provide adequate signal-to-noise ratio, given relatively modest levels of ambient response to wave and wind loading during the measurements.

**Figure 5.**
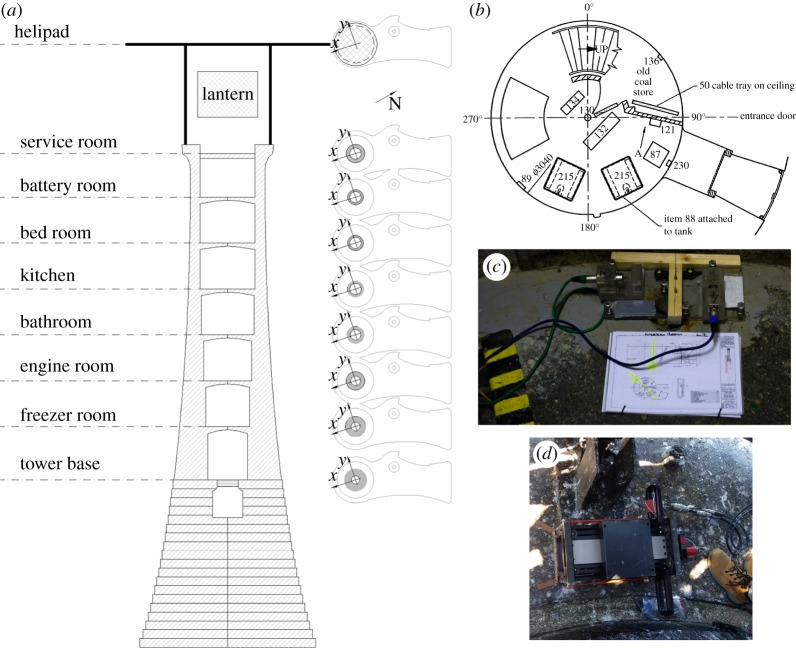
Wolf Rock lighthouse: (*a*) vertical section through tower, accelerometer angles, (*b*) sample internal layout, (*c*) QA 750 accelerometer placement in Freezer room and (*d*) APS 113 shaker at Service room. (Online version in colour.)

Along with uncertainty of signal-to-noise ratio came logistical constraints. The only access permitted for the measurements was by helicopter, which has limited payload and operates within specific weather limits. Additionally, all freight needs to be manhandled from helicopter hold via the helideck to the lantern gallery, limiting individual item weights. Reconnaissance was limited to internal layout drawings and prior experience with modal testing of Les Hanois lighthouse. 12 Honeywell QA 750 servo accelerometers were deployed in two consecutive sets to cover all nine available levels (L1–L9) of the lighthouse from entrance to helideck in orthogonal horizontal directions. The two directions (*x* and *y*) were chosen arbitrarily to make best use of internal features identified in floor plans enabling all sensors to be arranged in an almost vertical line and aligned, respectively, in *x* and *y* directions. Figure 5 shows the various levels and instrument deployment.

Table 1 details the measurements. Measurement duration was severely limited by time available on station between inbound and outbound flights, and included significant time setting out accelerometers and cables, and debugging shaker mechanical problems caused by strong vibrations in the helicopter hold.

**Table 1.** Wolf Rock measurement schedule.

set	levels	shaker direction	excitation	duration (s)
1	1,2,5,6,8,9	*x*	swept sine 3–30 Hz	900
1	1,2,5,6,8,9	*x*	swept sine 3.4–8 Hz	350
1	1,2,5,6,8,9	*y*	swept sine 3.4–8 Hz	400
1	1,2,5,6,8,9	—	ambient	900
2	3,4,7,8,9	—	ambient	80
2	3,4,7,8,9	*y*	swept sine 3.4–8 Hz	900
2	3,4,7,8,9	*x*	swept sine 3.4–8 Hz	630

**Figure 6.**
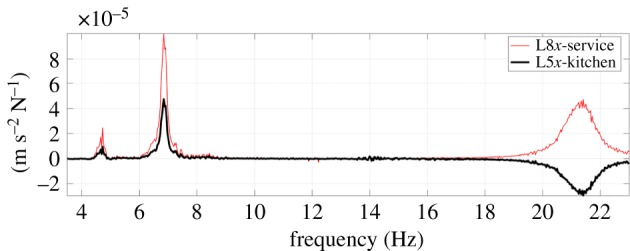
Wolf Rock imaginary part of FRF for 3–30 Hz swept sine. (Online version in colour.)

As planned, for each set, the shaker was aligned separately in *x* and *y* directions or switched off for ambient measurements. Swept sine excitation resulted in a better signal-to-noise ratio compared to random excitation for the broad range (3–30 Hz). Figure 6 shows the resulting frequency response function (FRF, ratio of response to input), with the antiphase around 21 Hz, indicating a higher order mode with a nodal point between L8 and L5. The signal-to-noise ratio was still relatively low, so the shaker force was focused in the range 3.4–8 Hz for better identification of the two lowest frequency vibration modes which were considered to be more relevant for dynamic response to breaking wave loads.

### (b) Experimental modal analysis

The FRF of figure 6 was calculated from force and response data using the H1 estimator [12] and clearly shows two strong structural modes below 10 Hz.

**Figure 7.**
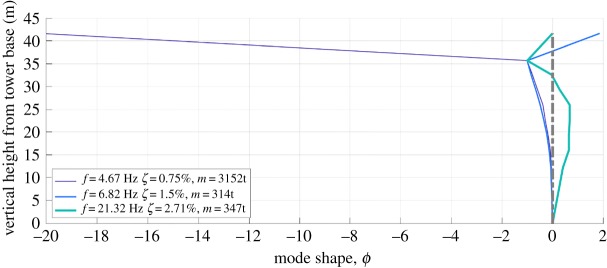
Mode shapes for *x* direction; *y* direction shapes are similar.

Two classical methods were used for experimental modal analysis. The global rational fraction polynomial (GRFP [13]) procedure was used to merge FRFs from set1 and set2 for both *x*-direction (shaking and response) and *y*-direction (shaking and response), allowing for identification of mode shapes. The circle fit (CFIT, [12,14]) single-mode system identification procedure was also applied to check the GRFP estimates, appearing to show high-quality identification for the fundamental mode with each shaker direction. The circle fit is a classical technique that fits to the ‘Nyquist’ plot of the real and imaginary components of the FRF. The natural frequency occurs where circle sweeps fastest through the data points which are at equal frequency intervals, the damping is available from the angle between data points at this frequency and the modal mass is derived from the radius of the circle. The method provides a powerfully visual but sometimes misleading way to estimate modal parameters. GRFP fits to the experimental data, an FRF for a predefined number of modes in a band (usually no more than three) with residuals for out of band modes. Both methods allow estimation of modal mass, and the values corresponding to point mobility at Service room (the shaker location) are shown with the mode shapes in figure 7. Note that the mode shapes for the first two modes shown are very similar within the tower but differ strongly in magnitude and sign for the helideck.

### (c) Operational modal analysis and effects of axial symmetry

The ambient response measurements were compromised by lack of time, so the set2 measurements were limited to a period at the end of a forced vibration measurement when the shaker had temporarily failed, hence a complete set of mode shapes covering both *x* and *y* directions was not available. The benefit of ambient response measurements is that the orientations (compass bearings) of the modes are not influenced by choice of sensor or shaker alignment.

**Figure 8.**
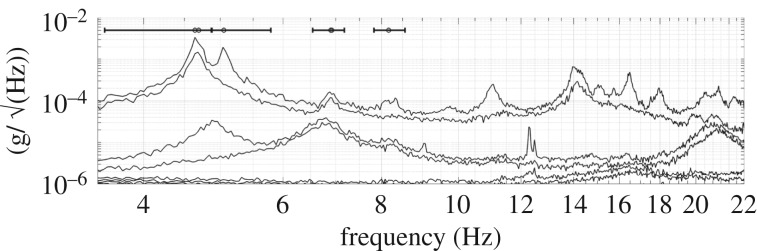
SVD of set1 ambient response data.

Figure 8 presents the singular value decomposition (SVD) of cross-spectral density (CSD) matrices generated from the 12 ambient response signals of set1, with frequency spacing 0.04 Hz. Near the resonance band of vibration modes, the rank of the CSD matrix at any frequency line is theoretically equal to the dimension of the subspace spanned by the ‘partial mode shapes’ (i.e. confined to measured degrees of freedom), which in the present case is equal to the number of contributing modes. In practice, the rank is usually identified by a sudden reduction in singular values to noise. Hence, SVD of the ambient response CSD matrix indicates the number of modes clearly through traces of singular values (SVs) peaking in a frequency band. The small circles above figure 8 indicate estimates of modal frequency and the error bars indicate bandwidth used for Bayesian operational modal analysis (BAYOMA [15]) for the range 0–10 Hz.

**Figure 9.**
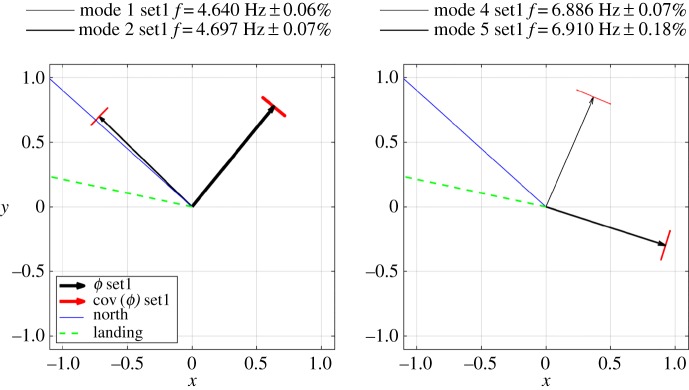
Uncertainty for first and second mode pairs and helideck mode shape orientations compared to true north and landing alignment.

There are very clearly two peaks (hence two modes) around both 4.7 and 6.8 Hz, plus two individual modes around 5 and 8.2 Hz, neither of which could be identified from FRFs. The orientation of these two mode pairs (1,2 and 4,5 in the sequence) identified using BAYOMA is shown in figure 9 together with the uncertainties (1 s.d.), while the bars on the arrow tips indicate the uncertainty in the mode shape alignment.

The directions within a mode pair need not necessarily be orthogonal unless they consider all degrees of freedom and are weighted by mass or stiffness. The closeness of mode pairs is a particular challenge for modal identification and reflects the degree of axial symmetry and the minor effect of mass and stiffness distribution variation, e.g. due to the landing. In terms of wave loading, it is unlikely that the weak mode directionality is a factor, but it is more relevant for validating the finite element model. In addition to the two pairs of modes, there are additional modes not clear in the FRFs that are suspected to involve little other than motion of the helideck.

### (d) Effect of helideck on modal properties

The more important features of the two pairs of modes which involve both helideck structure and masonry tower are that all four modes are zero-noded in the tower, i.e. they resemble the first mode shape of a cantilever. Figure 7 shows that for the lower pair, the helipad modal ordinate is in phase with the tower, while for the higher pair, the helipad ordinate is out of phase. The relative magnitude of the helipad/tower ordinates is close to 20 for the first pair, which is in fact the highest ratio measured among five helideck equipped lighthouses studied in STORMLAMP, all of which exhibit the same behaviour, to different degrees. Such behaviour is similar to that of a tuned mass damper [16], with the helideck acting as a lumped mass and stiffness [17] much smaller than the main structure but in similar proportion.

The important factor in terms of response to wave loading is the modal mass *m*. This has a strict definition in structural dynamics as the mass distribution (nominally expressed by mass matrix **M**) weighted by the square mode shape *ϕ*: *m* = *ϕ*^T^ **M***ϕ*.

Mode shapes have no units and since mode shape scaling is in principle arbitrary, modal mass can take any value. However, it is conventional in earthquake engineering and for some finite-element (FE) solvers to set mode shape scales such that the calculated value of *m* is one mass unit (e.g. 1 kg). A more physically meaningful mode shape scaling sets the value of the largest ordinate or the ordinate at a particular location on a structure where loading is likely to be applied to unity. This means that modal or generalized response parameters have direct physical interpretation, such as modal force and acceleration being actual values if a wave load is applied at the top of a lighthouse or for shaker testing. Specifically, figure 7 sets *ϕ* = 1 at the Service room (top of the masonry tower), while figure 9 sets *ϕ* = 1 at the helipad. The former is more appropriate for wave loading if the breaking wave impact is at Service room level, whereas in fact the effective point of application is much further down the tower. The lower modal mass of modes 4 and 5 means they will respond most strongly to wave impacts, and some sense of response to known load (or some sense of load for known response) can be obtained by scaling mode shape to unity at the wave impact point. Since the modal mass goes with square modal ordinate which decreases rapidly with height, the effective modal mass for breaking wave impacts will be very much higher than 314 t.

### (e) Response monitoring, winter 2017/2018

**Figure 10.**
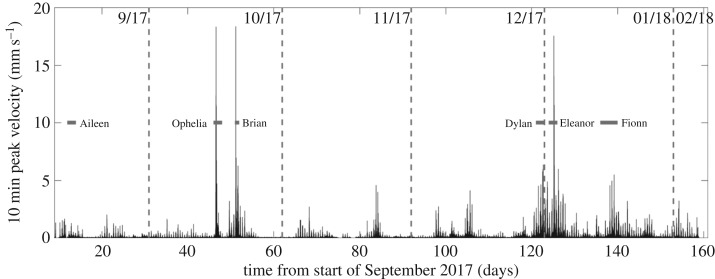
Velocity maxima during winter 2017–2018.

A remote logging system was installed at Wolf Rock on 7 September 2017 to acquire acceleration data from a single JA-70SA triaxial servo accelerometer installed in the Battery room (L7). While the logger still operates, a notable sequence of storms occurred during the period 9 July 2017 to 6 February 2018 including Storms Aileen (12–13 September 2017), Ophelia (formerly Hurricane Ophelia, 15–18 October 2017), Brian (20–22 October 2017) and Eleanor (2–3 January 2018). Acceleration signals due to wave impact events occasionally exceeded 0.2 g (approx. 2 m s^−2^) and were characterized by particularly strong response in modes 4 and 5, as expected due to the relatively low modal mass. Figure 10 presents the peak velocity values recorded during the period. Velocity is used because the wave impact loads are impulsive (i.e. short duration large force) which translates directly to instantaneous change of momentum followed by transient decays.

**Figure 11.**
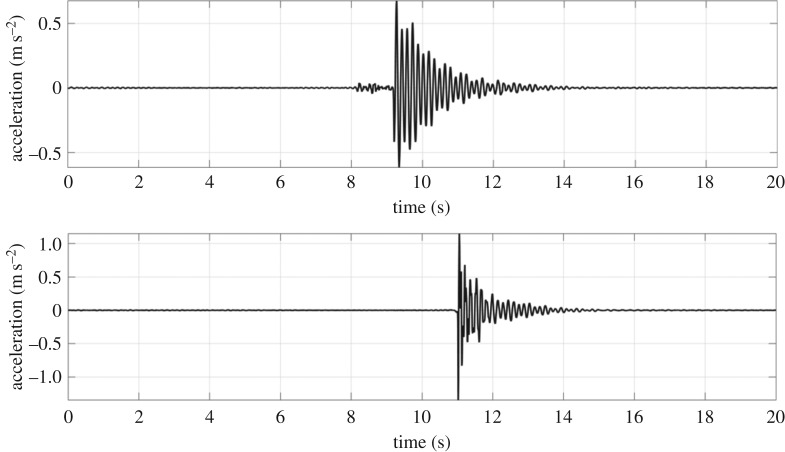
Acceleration signals corresponding to strongest velocities observed in Storms Brian (upper) and Ophelia (lower).

The raw acceleration signals corresponding to the maximum velocities for Brian and Ophelia are shown in figure 11. The Ophelia signal contains a relatively strong component of response in the third mode which is hardly evident in the velocities because integration reduces mode contributions in proportion to their frequencies.

## 4. Wave loading

Following characterization of the structure, the wave loading now needs to be determined. Statistical modelling of environmental extremes has a very practical motivation: structures need to withstand the environmental loading for the duration of their working life and, occasionally, even longer [18]; indeed, the Wolf Rock helideck is now beyond its specified design life. However, the application of extreme value (EV) theory to marine engineering, where the environment is particularly harsh, is problematic because: (i) high-quality data are difficult to obtain [19] and (ii) the accuracy of any methodology for EVs depends on the length of the recorded time series. Therefore, attention has shifted to statistical models applied to hindcast data where they have been adopted in numerous studies for the estimation of extreme wave conditions [20–22].

The present analysis is based upon a combination of two open access sea-state hindcast databases, both produced and managed by Ifremer (https://wwz.ifremer.fr/). The databases are HOMERE for the period from 1994 to 2012 [23] and NORGASUG for the period from 2013 to 2017, giving 24 years in total. Both databases cover the English Channel and the Bay of Biscay, and were obtained from the same Wave Watch 3 model, so the two datasets can be joined to give a longer one with uniform properties. An unstructured mesh has a resolution ranging from 200 m to 10 km, adapted at various scales from the open sea to the shoreline. The model outputs include hourly global wave parameters, e.g. the significant wave height (*H*_S_), peak period (*T*_P_), mean period, peak wave direction (*D*_P_) and mean wave direction for deep, intermediate and shallow water.

**Figure 12.**
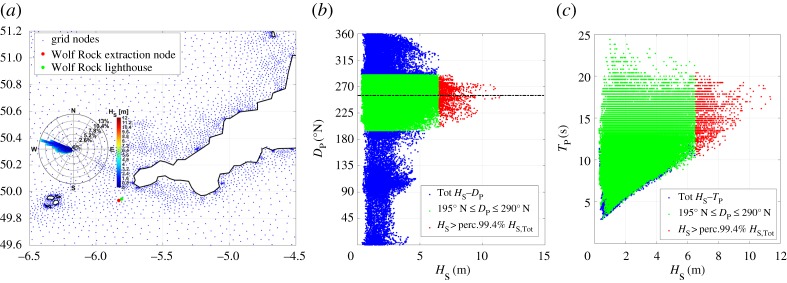
Wolf Rock lighthouse wave environment: (*a*) location, extraction node and wave rose, (*b*) peak direction versus significant wave height and (*c*) peak period versus significant wave height.

The selected extraction grid node is located southwest of the Wolf Rock lighthouse at a distance of 1.9 km from the lighthouse, with coordinates 49°56′4.7^″^ N–5°49′46^″^ W (figure 12*a*), in agreement with the main Atlantic north/western fetch and dominant wave direction (figure 12*b,c*). The water depth at this location is around 70 m. At this latitude, the oceanic nature of the sea state is characterized by a dominant sector between 195° and 290° N, and hence, data from this sector are the focus of the directional extreme analysis. The dominant direction, as calculated from the median values for *H*_S_ > 99.4 percentile, is equal to 255° N (figure 12*b*), clearly showing the sheltering effects of the Isles of Scilly. The severe nature of the largest waves is also highlighted by the associated *T*_P_ that are always larger than 10.5 s (figure 12*c*).

### (a) Bayesian inference for extreme wave height analysis

The extreme wave heights are estimated based on the methodology presented by Antonini *et al*. [24] which considered the Fastnet lighthouse. However, the present analysis considers non-stationarity through a linear trend in the statistical model parameters in order to describe the minimal increasing long-term trend in the extreme wave heights previously identified by Bricheno & Wolf [25] and Aarnes *et al*. [26]. The adopted method involves a non-stationary Bayesian EV analysis of the directional wave heights exceeding a threshold: the peak over threshold method. Furthermore, it is assumed that the number of threshold *u* exceedances in the investigated time interval follows the Poisson distribution. Thus, the selected extreme events should occur randomly in time according to the Poisson process, with annual mean *νT*, where *ν* is the event rate (yr^−1^) and *T* = 1 yr. Events should be sufficiently far apart to be independent, and the exceedances to *u* should have an approximate generalized Pareto distribution (GPD)

4.1 $$G_{\left(y,\sigma ,\xi \right)}=\left\{ \begin{array}{ll} 1-{\left(1+\xi \frac{y}{\sigma }\right)}^{-(1/\xi )}; & \xi \neq 0\\ 1-\exp\left(-\frac{y}{\sigma }\right); & \xi =0, \end{array}\right.$$

where ***y* **= *y*|*_y_*_>*u* _− *u* is the exceedance by *y* of the threshold *u*, *ξ* is the shape parameter and *σ* > 0 is the scale parameter. The combination of the Poisson model for frequency and the GPD model for intensity can be expressed in a form compatible with the generalized extreme value distribution (GEV) for annual maxima provided the following equations are respected ([19,27,28])

4.2 σ=ψ+ξ(u−μ)

and

4.3 $$\nu ={\left(\frac{\xi (u-\mu )}{\psi }\right)}^{-1/\xi },$$

where *ψ* > 0 and *μ* are the GEV's scale and location parameters, respectively.

**Figure 13.**
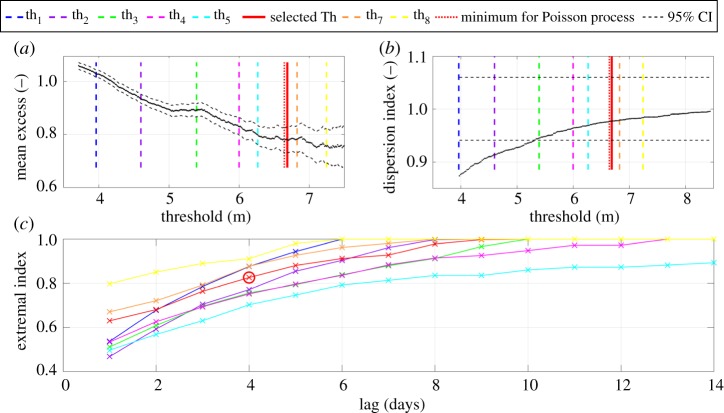
(*a*) Mean residual life plot, (*b*) dispersion index and (*c*) extremal index for combination of all eight investigated thresholds and 14 declustering time spans.

The GPD–Poisson model presents the critical problem of selecting the appropriate threshold and minimum declustering time span, *n*. Following Antonini *et al*. [24], the threshold and time span selection are based on three diagnostic plots. The mean residual life plot is shown in figure 13*a*, the dispersion index proposed by Cunnane [29], shown in figure 13*b* and the extremal index first applied by Ferro [30], shown in figure 13*c*. The tested threshold values from th_1_ to th_5_ are clearly violating the Poisson assumption being smaller than the minimum required value shown in figure 13*a,b*. Moreover, the level of dependency of the identified peaks hardly respects the assumption of independent events, requiring a relatively large time span for an effective declustering process (figure 13*c*).

**Figure 14.**
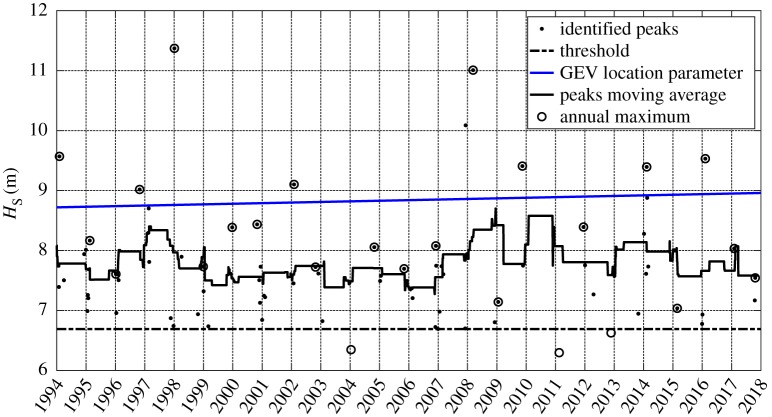
Annual maxima series (time interval 1 July to following 30 June), exceedances over the 99.4% *H*_S_, in blue, the estimated location parameter *μ*_(*t*)_, in black, the moving average obtained with 2.1 years moving window on the identified threshold exceedances. (Online version in colour.)

Eight threshold values are investigated as well as 14 declustering time spans, with conclusions similar to those achieved by Antonini *et al*. [24]. The smallest tested threshold value above the Poisson limit, i.e. 99.4 percentile corresponding to 6.69 m, and a declustering time span equal to 4 days, is the best compromise between the amount of data and the independence of the selected storms. The stated parameters lead to a dataset of 61 peaks, i.e. 2.54 events per year as presented in figure 14.

The model is expressed in terms of the GEV's parameters using the time-dependent version of equation (4.3) to relate the GPD–Poisson parameters to the equivalent GEV parameters. The analysis seeks to detect a long-term trend allowing the location parameter to be time-dependent (*μ*_(*t*)_) [31], using the linear parametric expression

4.4 $$\mu_{\left(t\right)}=\mu_{1}\cdot t+\mu_{0},$$

where *t* is the time (in years), and *μ*_1_ and *μ*_0_ are the adopted parameters resulting from the Bayesian process equal to 0.01 m yr^−1^ and 8.72 m, respectively, as shown in figure 14. The detected trend is in agreement with the upward trends along the Atlantic coast of Europe described by Bromirski & Cayan [32] and it is also within the range of the upward trend detected by Wang & Swail [33] for the winter extreme waves along the northeast North Atlantic. The basic idea of the adopted non-stationary approach is to hold the probability of occurrence constant in time, but allowing the return value, i.e. *H*_S_, to vary from one time period to the next. Through the manuscript, the effective return level is used to indicate what return level should be used, within the investigated time interval (i.e. 2017–2067), to have the same risk [34]. Extrapolation into the future assumes a continuous linear trend in extreme wave heights, as has been detected for the available dataset, though neglecting the decadal variation due to the NAO fluctuation and the other cyclical atmospheric modes highlighted by Santo *et al*. [35]. The omission of these further factors will increase the uncertainty of the 2067 design wave heights.

The Bayesian technique is used to infer the distribution parameters; furthermore, the uncertainty estimates for the effective return level are provided by means of Bayesian-based Markov Chain Monte Carlo approach (MCMC) as already proposed by Stephenson & Tawn [36].

### (b) Priors distribution

**Figure 15.**
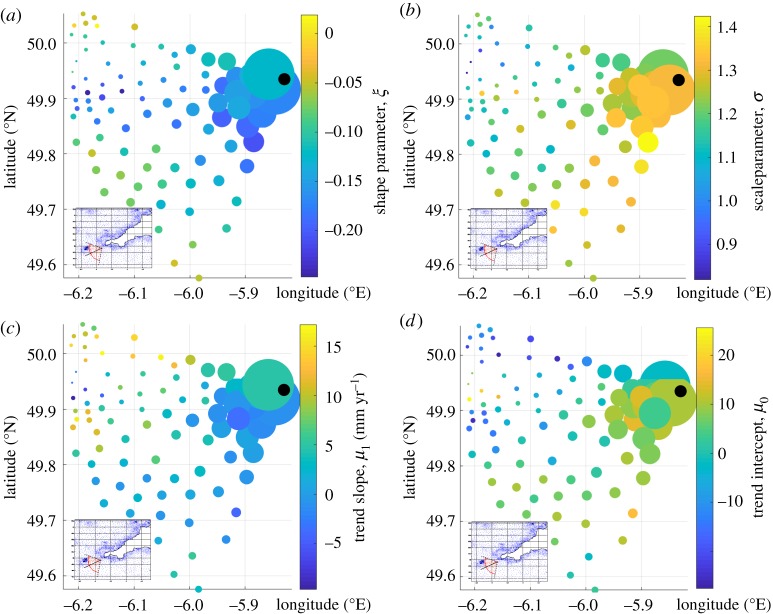
(*a,b*) Estimated shape and scale parameters and (*c,d*) estimated linear trend parameters for each of the 94 grid points. The size of each dot is proportional to the weight used to calculate the mean and standard deviations of the prior distributions.

The use of hindcast database allows access to a vast amount of data and information. The adopted Bayesian model aims to incorporate the information provided by the surrounding grid nodes, selected on the basis of the dominant fetch, into the estimation of the effective return level. To this end, 94 grid points, covering an area between 190 and 290° N for a maximum distance of 30 km from the Wolf Rock extraction point, have been used to describe the prior distributions for the model parameters (figure 15). In the adopted method, the idea is to estimate the weighted mean value and standard deviation for the prior normal distributions of the GPD's shape (*ξ*) and scale (*σ*) parameters as well as the intercept (*μ*_0_) and the slope of the linear trend (*μ*_1_) from the surrounding grid points. Figure 15*a,b* shows the results from the classical GPD fitting procedure as a function of location, while figure 15*c,d* shows the results from the linear regression applied to the identified storm peaks.

In figure 15, the black dot identifies the location of the Wolf Rock extraction point. The other points show the value of the estimated parameters (colour of the points), while the size is proportional to their weight used for the calculation of the mean and standard deviation of the prior normal distributions. Two parameters are used to compose the weight vector: the inverse of the distance between the selected point, among the 94 identified ones, and the Wolf Rock extraction point and the inverse of the norm between the two vectors defining the *H*_S_ dominant sector of the extraction point (i.e. [195; 255; 290]) and the selected ones for the prior distributions, table 2.

**Table 2.** Mean and standard deviation of the prior distributions.

	mean	s.d.
shape parameters (*ξ*)	−0.1512	0.0333
scale parameters (*σ*)	1.2684	0.0684
trend slope (*μ*_1_)	0.0014 (m yr^−1^)	0.0034 (m yr^−1^)
trend intercept (*μ*_0_)	5.1322 (m)	6.8506 (m)

### (c) Design wave conditions

**Figure 16.**
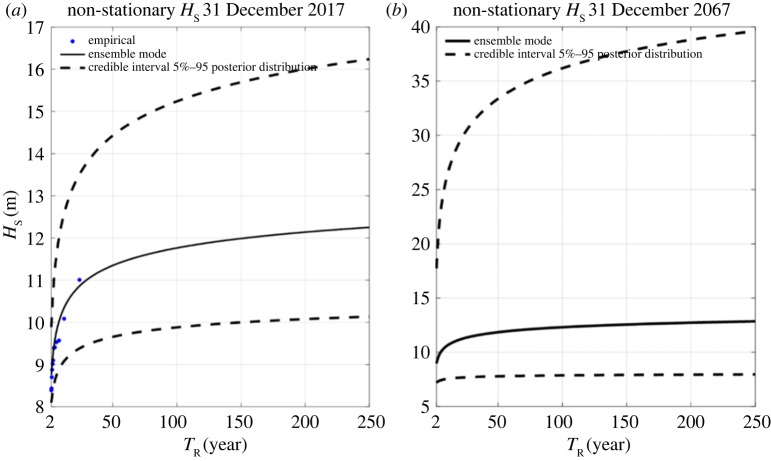
Identified design values and related credible intervals at the end of the observation period: (*a*) 31 December 2017 and (*b*) 31 December 2067.

The design wave is obtained using a posterior distribution by means of Differential Evolution Markov Chain Monte Carlo (DE-MC) algorithm [37]. A total of 10 000 realizations of the prior and posterior distributions are generated, for 10 parallel chains. The potential scale reduction factor [38] is used to evaluate the convergence, while the acceptance rate is adopted as the indicator of the goodness of the mixing property of the DE-MC chains [39]. The classical definition of return level, *T*_R_, and non-exceedance probability, *p*, are adopted in order to estimate the time-dependent extreme quantile, *q_p(t)_*. The generated ensemble is the basis of the hazard parameters distribution, i.e. *H*_S_, and related credible interval. Moreover, since this study concerns the evaluation of the structure survivability, the design values are required; hence, the most probable values of the ensemble are used as the final return level values [40] (figure 16).

The model parameters will then be used to estimate the non-stationary return levels as shown in equations (4.5) and (4.6), where *k* indicates the order of the percentile used within the location posterior distribution. Owing to the critical nature of the lighthouse and the model uncertainties (when used beyond the observations), 95 percentiles of the *μ*_(*t*)_ values in historical observation are used

4.5 $$\begin{array}{rl} q_{p(t)} & =\left({\left(-\frac{1}{\ln p}\right)}^{\xi }-1\right)\cdot \frac{\psi }{\xi }+{\tilde{\mu }}_{\left(t\right)} \end{array}$$

4.6 $$\begin{array}{rl} {\tilde{\mu }}_{\left(t\right)} & =Q_{k}(\mu_{t1},\mu_{t2},\mu_{t3}\ldots \mu_{tn});\mu_{\left(t\right)}=\mu_{1}\cdot t+\mu_{0} \end{array}$$

**Figure 17.**
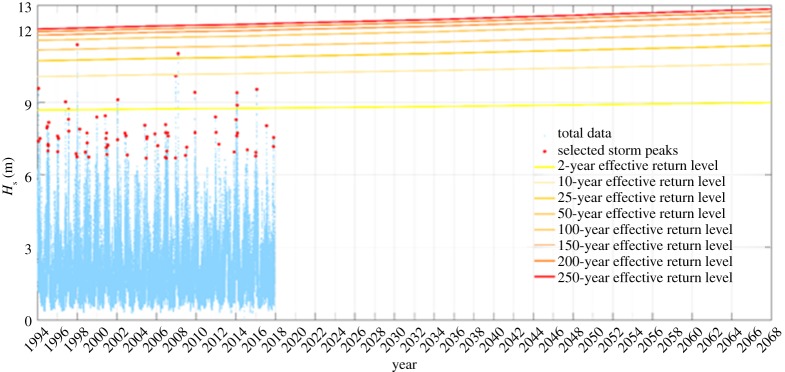
Effective return levels under the non-stationary assumption at the Wolf Rock extraction point.

Figure 17 shows the effective return levels as a function of the time covariate (equation (4.4)) for the Wolf Rock extraction point. Note that the return levels vary over time such that the probability of occurrence remains constant.

A classical three-parameter power law presented in equation (4.7) is used to describe the *T*_p_ associated with the predicted extreme *H*_S_ (e.g. [6,22,41])

4.7 $$T_{P}=a\cdot H_{S}^{b}+c.$$

A linear regression of the measured peak periods versus the identified storm peaks yields: *a* = −169.7 (−1497, 1158), *b* = −1.63 (−6.89, 3.64), *c* = 20.97 (3.09, 38.84), where the values in parentheses indicate 95% lower and upper bounds parameters estimation. The final results of the extreme wave analysis procedure are two set of wave states to be used during the survivability assessment of the lighthouse. The first set of non-stationary conditions refers to the end of the observation time series, i.e. 31 December 2017, while the second is a statistical forecast based on the detected trend of the available data and it refers to 31 December 2067, i.e. 50 years after the end of the observation.

### (d) Impulsive wave loading definition

The identification of the impulsive wave loading on the lighthouse comprises five steps as described in detail by Antonini *et al*. [24]:

(i) Characterization of the offshore extreme wave climate to propagate to the lighthouse location (from the last subsection).
(ii) Estimation of the significant wave height at Wolf Rock location (*H*_S,L_) by means of Goda's method [42]. The required data, i.e. local sea bed slope and water depth, are collected from the INFOMAR website (https://jetstream.gsi.ie/iwdds/map.jsp), the Navionics website (https://webapp.navionics.com/) and from the archive drawings provided by Trinity House, resulting in values equal to 1/3 and 35 m, respectively. The selected point at which the wave height was calculated corresponds to a 35 m water depth, located approximately 70 m from the lighthouse, i.e. around 1/3 of the significant wavelength for the 250-year return period.
(iii) Description of site-specific wave height distribution. Here, we use the composite Weibull distribution (CWD) proposed by Battjes & Groenendijk's [43] and select *H*_0.1%_ as the main design parameter. It is worth mentioning that the effect of the reduced local water depth is not effective in reducing the local wave condition due to the relatively large value even without considering the tide and sea-level rise (SLR) effects. Indeed, the ratio between the 250-year wave heights and the water depth is between 0.68 and 0.71, well below the criteria of shallow water breaking condition proposed by several authors, e.g. [44–47]. Moreover, the bathymetry offshore of the Wolf Rock pinnacle does not show any relevant or abrupt variation of the bottom that might induce breaking of the waves before the rock. SLR is estimated to be 0.36 m at the Wolf Rock location, using the latest predictions from UKCP18 [48].
(iv) Calculation of the crest elevation with respect to the still water level (*η*_b_) [49].
(v) Application of Wienke & Oumeraci's [50] method to describe the total slamming load on the varying diameter lighthouse and the spatial pressure distribution. The total horizontal load is considered spatially uniform for both the horizontal and vertical direction, while the affected frontal sector is limited to ±30° of the selected wave direction, i.e. *D*_P_ = 255° N. The main assumptions fundamental to the calculation are in terms of: the curling factor (related to the extent of the surface elevation (*η*_b_) of the breaker that contributes to the impact) which is assumed to be 0.46 as proposed in [50]; the fluid velocity within the wave crest that is assumed to be equal to the wave celerity calculated according to the solitary wave theory proposed by Grimshaw [51]; and an assumption that the wave breaks directly in front of the lighthouse. These assumptions generate an overall conservative approach that aligns with the aim of this manuscript, focused on the survivability assessment of the Wolf Rock lighthouse.

These steps produce a set of wave characteristics describing the extreme wave climate at the Wolf Rock location and the associated loading conditions. Moreover, the average radius, the upper and lower points describing the lighthouse area affected by the wave impact, as well as the total impulse of the loading case are presented in table 3 for 2017 conditions and in table 4 for the forecasted conditions. They are also indicated in figure 20. It is clear that there is not a large difference in the total impulse for both the current extreme wave climate and the predicted one. The main reason is related to the different lighthouse area affected by the impact. For the current extreme wave conditions, the impacts happen around a lower area of the structure, while for the future ones, the impact areas are slightly higher, but the load calculation considers the average radius which reduces with the increase in wave heights and consequently with the intensity of the pressure. Thus, the pressure intensity and size of the radius compensate for each other.

**Table 3.** Design wave climate and related load conditions 31 December 2017.

*T*_R_ (year)	*H*_S_ (m)	*T*_P_ (s)	*T*_S_ (s)	*H*_S,L_ (m)	*H*_0.1%_ (m)	*η*_b_ (m)	upper (m)	lower (m)	radius (m)	impulse (kN s)
2	8.76	16.0	14.2	8.76	16.55	12.37	12.37	6.68	3.62	1311.71
10	10.21	17.1	15.1	10.21	19.49	14.40	14.40	7.78	3.46	1415.64
50	11.35	17.7	15.7	11.35	21.85	15.97	15.97	8.62	3.34	1480.55
100	11.77	17.9	15.8	11.77	22.72	16.53	16.53	8.93	3.30	1498.45
200	12.14	18.0	16.0	12.14	23.51	17.04	17.04	9.20	3.27	1517.67
250	12.25	18.1	16.0	12.25	23.75	17.19	17.19	9.28	3.26	1522.56

**Table 4.** Design wave climate and related load conditions 31 December 2067.

*T*_R_ (year)	*H*_S_ (m)	*T*_P_ (s)	*T*_S_ (s)	*H*_S,L_ (m)	*H*_0.1%_ (m)	*η*_b_ (m)	upper (m)	lower (m)	radius (m)	impulse (kN s)
2	8.98	16.2	14.3	8.98	16.99	12.68	12.68	6.85	3.56	1307.30
10	10.58	17.3	15.3	10.58	21.82	15.96	15.96	8.62	3.31	1456.63
50	11.85	17.9	15.9	11.85	22.89	16.64	16.64	8.99	3.27	1483.62
100	12.31	18.1	16.0	12.31	23.85	17.26	17.26	9.32	3.23	1503.86
200	12.73	18.3	16.2	12.73	24.73	17.82	17.82	9.62	3.19	1520.27
250	12.85	18.3	16.2	12.85	25.00	17.99	17.99	9.71	3.18	1524.91

## 5. Structural analysis

### (a) Numerical modelling approach

**Figure 18.**
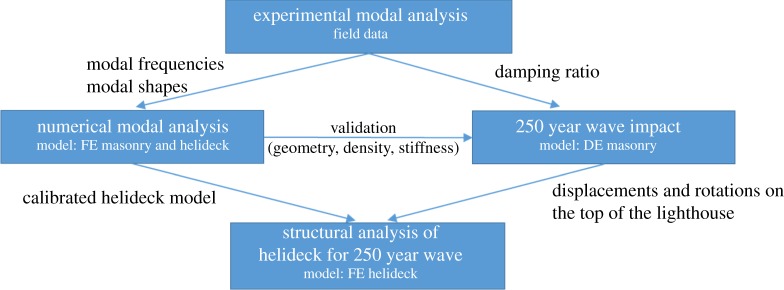
Structural analysis workflow.

The structural analysis presented in this paper aims to investigate the structural response of the helideck to extreme waves impacting the lighthouse. The workflow of the model calibration and structural assessment is presented in figure 18.

The modal frequencies and shapes identified in the experimental modal analysis are used for calibrating the finite element (FE) model of the lighthouse, consisting of the granite masonry structure and the helideck. Research has shown that the lighthouse structure, which is expected to exhibit rocking behaviour due to lack of bonding between horizontal courses, has to be analysed using a discontinuous model [52,53] to account for the substantial damping effect generated in the rocking motion. Therefore, a discrete element (DE) model of the granite structure is used to determine the response of the lighthouse to the 250-year return period wave impact scenarios. To analyse the effect of the wave impacts on the structural response of the helideck, a submodel of the helideck is used. For this analysis, the displacements and rotation recorded on the top of the masonry structure (i.e. the base of the helideck) from the DE analysis are used as input data for an FE helideck model. Finally, the FE helideck model is used to identify potentially critical areas where damage or failure is expected. This substructuring approach is followed due to extensive computational demand of an FE analysis with discontinuous dovetailed blocks. Moreover, the DE code which is very efficient for modelling such discontinuous and complex structures has limitations regarding the modelling of the steel helideck.

### (b) Finite-element model description and calibration

The commercial software Abaqus [54] is used for the FE modelling. Continuous and homogeneous material properties are used for both the lighthouse body and the helideck structure. For the granite masonry body, we use a structured and swept FE mesh with 92 386 three-dimensional 8-node reduced integration linear brick elements (C3D8R). The helideck is modelled with 9127 Timoshenko beam elements (B31) that allow for transverse shear deformation [54]. The FE model uses the geometry of the masonry blocks obtained from archive drawings of the construction [55], and the metal profiles of the helideck taken from the structural design documentation [56]. Based on the field test findings presented in §3*b*, Rayleigh damping of *ζ* = 0.75% at 4.67 Hz (*α* = 0.22007, *β* = 0.00026) is applied to the whole model.

The masonry density is taken as 2643 kg m^−3^, which corresponds to coarse-grained Cornish granite. Such material properties were used for the calibration of the historic Fastnet Rock lighthouse, built with the same typology as the Wolf Rock lighthouse [53,57]. The masonry modulus of elasticity is taken to be 37 GPa. The helideck is built with Grade 43C steel, defined by BS 4360:1990 [58], which is equivalent to the modern S275 defined by Eurocode 3 [59]. The density and modulus of elasticity are assumed to be 7850 kg m^−3^ and 200 GPa, respectively. An isotropic hardening constitutive law is taken for the nonlinear behaviour of steel. For this type of steel, Eurocode 3 [59] gives: yield stress *f*_y_ = 255 MPa, ultimate stress *f*_u_ = 410 MPa and ultimate plastic strain *ϵ*_u_ = 0.2.

**Figure 19.**
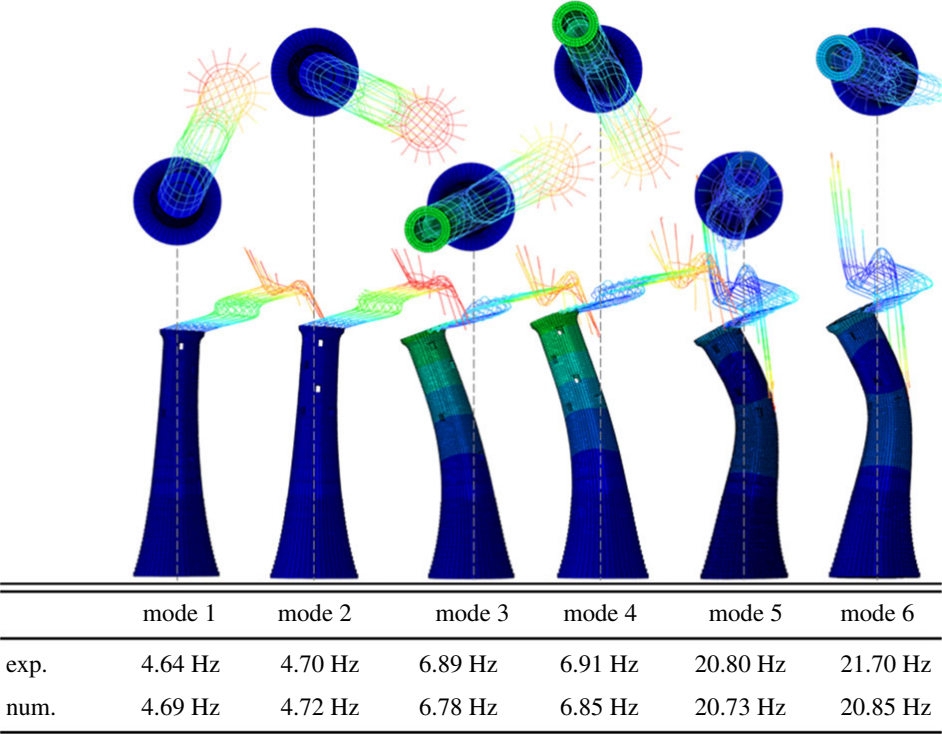
Numerical modes of vibration, and numerical versus experimental modal frequencies. (Online version in colour.)

The numerical modal analysis results of the calibrated FE model are shown in figure 19. A close fit, in terms of mode shapes and modal frequencies, is obtained between the numerical and experimental results. The error is less than 1.5% for the first five modal frequencies and around 4% for the sixth mode. The FE model correctly captures the behaviour revealed by the experimental modal analysis. Figure 19 shows that for the first four modes, the masonry tower behaves like a cantilever with zero-node mode shape, while the helideck is in phase for the first couple of modes and out of phase for the second couple. For the fifth and sixth mode, similar to the findings of the on-site dynamic identification, the masonry tower behaves like a cantilever with a single-node mode shape and the helideck is in phase with the lower part of the tower.

In order to study the influence of the helideck on the dynamic characteristics of the structure, two more FE modal analyses are carried out. The first is the modal analysis of the masonry structure without the presence of a helideck. This yields the first pair of frequencies at 6.65 and 6.73 Hz, and a second pair at 20.93 and 21.06 Hz. The second analysis considers only the helideck structure, for which the first pair of modal frequencies are calculated to be 4.90 and 4.95 Hz.

### (c) Wave force time-histories and impact areas

**Figure 20.**
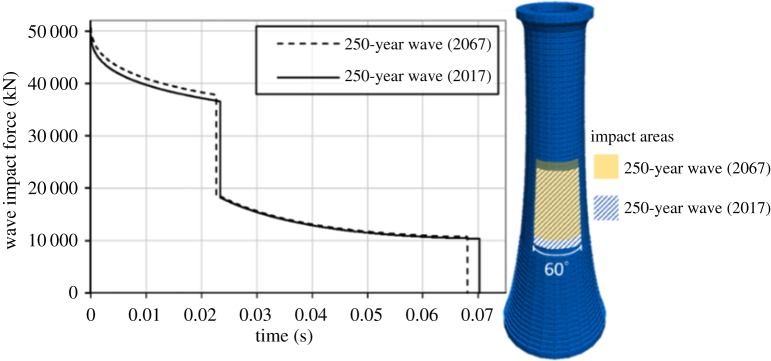
Force time-histories and impact areas on the DE model for the 250-year return period waves.

The 250-year return period waves have been calculated both for the 2017 series and the statistical forecast of 2067, as described in §4d. For the 2017 series, the total impact duration is 0.070 s, and the maximum horizontal impact force, at *t* = 0, is 49 510 kN (dashed line in figure 20). The forces are applied between the 23rd and the 40th course. For the 2067 series, the maximum impact force increases to 51 149 kN, while the impact duration decreases slightly to 0.068 s (continuous line in figure 20). The impact area is two courses higher, i.e. between the 25th and the 42nd course. The impact areas and the time-history of the total force that the impulsive waves apply on the structure are presented in figure 20. Note that the duration of both wave impacts is significantly shorter than the first pair of modal periods, i.e. between 0.21 and 0.15 s.

### (d) Helideck structural assessment for wave impacts

The lighthouse is built with granite interlocked blocks and is expected to present local uplift and opening of the horizontal joints for intense impacts [52]. It has been shown that a continuous model is not capable of yielding satisfactory results, underestimating the amplitude of structural response in terms of displacements [53]. Also, continuous elastic models, due to high stiffness, induce a higher frequency content structural response and consequently higher values of acceleration and velocity. Therefore, the DE method is used herein for analysing the structural response of the granite structure to wave impacts. The DE is efficient for reproducing structural response of rigid bodies that are characterized by large displacements and separation between blocks. A three-dimensional DE model of the masonry structure is developed with the commercial software 3DEC [60].

Each course of the DE model consists of 12 rigid blocks. The vertical keys are also modelled, hence impeding large sliding unless significant uplift takes place. The Coulomb friction law is implemented for the joints between blocks with zero cohesion and an angle of friction of 30°. Moreover, the joint is given normal stiffness of 7.31 × 10^10^ Pa m^−1^ and shear stiffness of 5.48 × 10^10 ^Pa m^−1^. Additional mass is added to the top course in order to account for the mass of the lantern and helideck which are not explicitly modelled at this stage.

**Figure 21.**
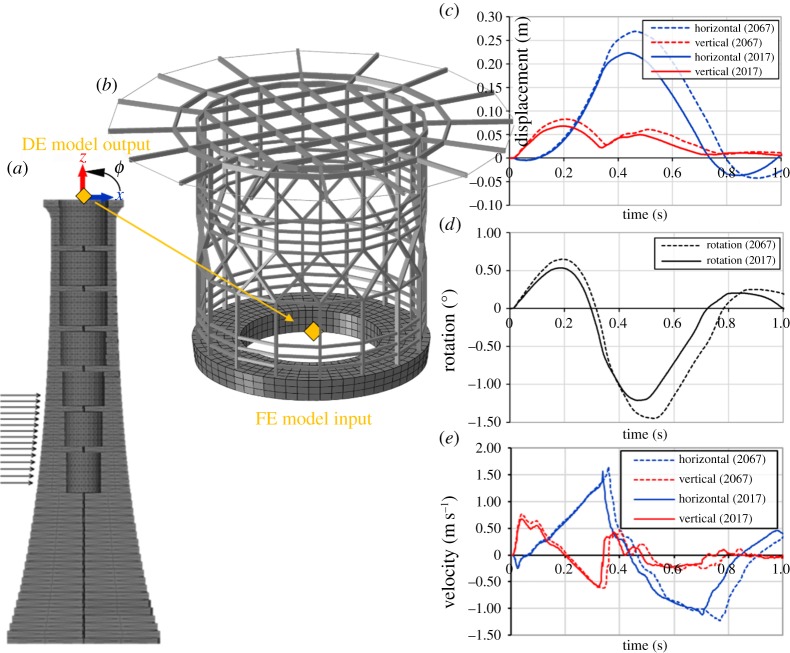
(*a*) DE model section and control point on the top course; (*b*) FE helideck submodel and application of displacements and rotation yielded by the DE model; (*c*) displacements; (*d*) rotation response and (*e*) velocity response time-histories on the top course.

For the structural assessment of the helideck for extreme wave impacts, the hybrid approach presented in figure 21 is adopted. The DE time-history analysis outputs, i.e. displacements and rotation obtained on the top course (figure 21*a*), are used as input data for the FE analysis of the helideck submodel (figure 21*b*). For the 2017 series, the DE analysis revealed maximum horizontal and vertical displacements at the centre of the top course of 0.223 and 0.068 m, respectively (figure 21*c*). The maximum rotation around the same point is equal to 1.21° (figure 21*d*). For the 2067 forecast, the horizontal and vertical displacements increase to 0.269 and 0.083 m, whereas the maximum rotation is equal to 1.45°. Therefore, it is shown that the elevation of the impact area and the slight increase in impact forces amplify the structural response of the masonry structure. Note that the maximum horizontal velocity yielded by the DE analysis is around 1.5 m s^−1^ for both series (figure 21*e*), which is around 75 times higher than the peak velocity recorded during the 2017–2018 storms (figure 10).

The structural analysis presented herein focuses on the level of axial tensile reaction forces at the base of the gallery posts, which would have a tendency to pull out the steel connectors in the granite body or fracture the bolts. Moreover, the overall stress level at the helideck steel structure, which can cause yielding and failure, is presented.

**Figure 22.**
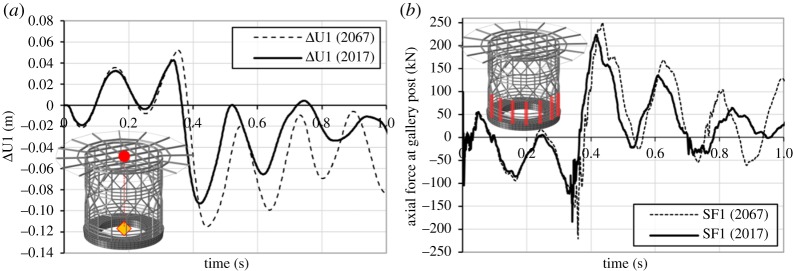
(*a*) Differential horizontal displacement between centre of helideck base (yellow) and control point on the top (red) and (*b*) response time-history of gallery vertical post with the highest axial forces.

Figure 22*a* shows the differential horizontal displacement, after removing the displacements due to rotation, between the centre of the base and a control point on the top of the helideck. The analyses yield maximum absolute values of horizontal deformation equal to 0.093 and 0.115 m for the 2017 and 2067 series, respectively. In both cases, the helideck oscillates with frequency content of around 4.5–5.5 Hz. Figure 22*b* shows the response time-history of the gallery vertical post that presents the highest axial reaction forces. The analyses reveal high levels of tensile reaction forces. The 2017 series wave causes tensile reaction equal to 223.5 kN, which becomes 250.7 kN for the 2067 forecast, or an increase of 12%. Although these forces do not cause tensile failure of the gallery posts' steel section, they can pose a threat to the connecting bolts at the base of the helideck and the encastrated bars.

**Figure 23.**
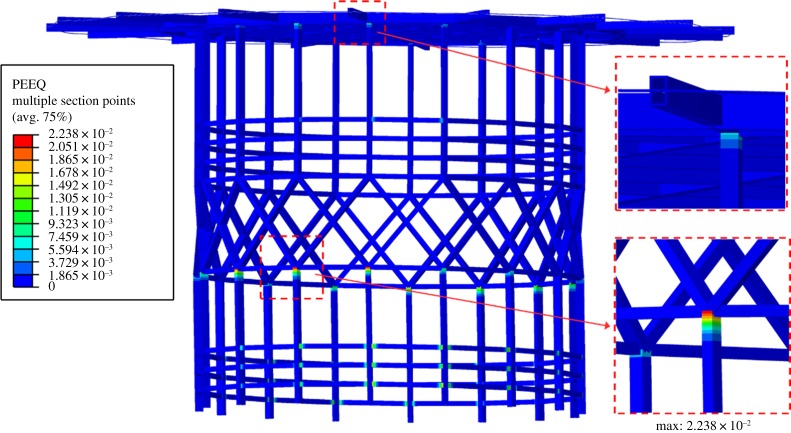
Plastic strains on helideck steel structure for the 250-year return period wave impact scenario of the 2067 forecast.

The numerical analysis also reveals that modest local yielding of the steel structure due to tensile stresses is expected. Disregarding the horizontal railings that are secondary structural elements, the most problematic areas are the tops of the vertical gallery posts. Figure 23 shows the numerical analysis results for the 250-year return period wave impact scenario of the 2067 forecast. The analysis of the 2017 scenario present the same yield areas but with lower levels of plastic strain. For the 2067 forecast, the maximum plastic strain increases from 0.0130 to 0.0238, or an increase of 83% compared to the 2017 values. These plastic strain levels correspond to around 7 and 12% of the assumed plastic strain capacity, for the 2017 and 2067 scenarios, respectively. Therefore, although the impact of the 250-year return period wave for both scenarios is expected to cause some limited damage and permanent deformation, the tensile rupture of the steel sections caused by a single wave of this intensity is not likely.

## 6. Discussion and conclusion

In this paper, we have traced the developments at Wolf Rock over a period of nearly 200 years, from rudimentary beacons that were destroyed by huge seas to the current rock lighthouse now topped by a helideck and shrouded by PV panels, typifying the new vernacular of UK offshore lighthouses. These structures, far from belonging to a bygone era, are intended to be kept as physical aids to navigation through the twenty-first century. To assess the future survivability of the lighthouse and the helideck, a sequence of investigations was undertaken. Ultimately, a structural model was required, but it needed validating with field data and estimates of wave loading, both for today's climate but also for future conditions.

The challenging modal tests produced data for validation of the structural model, believed to be the first full-scale vibration test of a rock mounted lighthouse providing direct estimation of modal mass, and defining mode frequencies, damping ratios and mode shapes (with horizontal alignment) with mean and variances. The tests produced clear responses at about 4.7, 6.8 and 21.3 Hz, identified in both perpendicular directions. Modal shapes for the lower modes were similar within the tower but very different at the helideck level. Furthermore, the mode pairs involving the helideck structure and tower resemble a cantilever mode shape; this was also borne out by the FE model. The identification of the three associated modal masses (3152, 314 and 347 tonnes) will enable the inference of the applied load (i.e. the impacting wave), according to the measured structural response. This is possible using the logging system installed on the lighthouse, which has recorded accelerations of the order of 0.2 g, not dissimilar to modest earthquakes.

The load distribution on the lighthouse required complex statistical techniques to be developed. A Bayesian method with informative prior distributions was defined from information provided by the surrounding numerical model grid points at Wolf Rock. Prior distributions were defined on the basis of the wave climate, i.e. the information is weighted according to the similarity between the wave climate of the location and the points used for the prior distributions. A statistical forecast of the return period based on a minimal upward trend, deduced from available data, was then converted into a load distribution for the lighthouse.

A structural assessment of the interlocking masonry lighthouse with mounted steel helideck was then analysed with a hybrid approach that combined DE and FE modelling. The DE modelling was based upon meticulous modelling of the interlocking blocks of the lighthouse. Predictions of maximum horizontal tower displacements were 0.223 and 0.269 m, respectively, for the 250-year return period wave loading in 2017 and 2067. This is a 20% increase over the 50-year period, and considerably larger than the fraction of a millimetre displacement measured on the Eddystone [1]. Vertical displacements were of the order of about one-third the horizontal displacements, but still not insignificant. The horizontal displacement of the helideck with respect to the tower was 0.093 and 0.115 m for the 1 : 250 year extreme wave for 2017 and 2067 conditions. These were associated with high tensile reaction forces in the gallery posts of 223.5 and 250.7 kN, respectively, large enough to damage bolts at the base of the helideck and the encastrated bars. Finally, strain associated with these tensile stresses, although showing a significant increase from 2017 to 2067 conditions, is still only 12% of the assumed plastic strain capacity.

Trinity House depends on these lighthouse helidecks for the same reason they were constructed in the first place: to enable rapid deployment when equipment breaks, now that the stations are unmanned. The situation is now exacerbated by the fact that boat skills and knowledge of landing people on the lighthouse has been lost since helidecks have been in operation. The identification of structural elements most vulnerable to high stress/strain arising from the structural modelling is important as it will inform Trinity House's visual inspection surveys. This will provide operational confidence for the lighthouse authorities into the future. Finally, this comprehensive investigation has demonstrated techniques that can be applied to other coastal and offshore structures. For example, the historic masonry seawall and breakwaters that are common around European coastlines may be modelled and monitored in similar ways to assess their condition under wave loading. Also, more contemporary structures, such as gravity-based foundations for offshore wind turbines, share similarities in their physical configurations [61] and hence lend themselves to wave modelling techniques developed in the STORMLAMP project.

## Data accessibility

Data relevant to vibration tests, wave statistics and structural modelling can be accessed at Pearl doi:10.24382/pr8p-g562.

## References

[1] Raby A, Bullock GN, Banfi D, Rafiq Y, Cali F 2015 Wave loading on rock lighthouses. Proc. Inst. Civ. Eng. Marit. Eng. 169, 15–28. (10.1680/jmaen.15.00002)

[2] Williams T 1923 Life of William Douglass M.Inst.C.E. Printed for private circulation.

[3] Douglass JN 1871 The wolf rock lighthouse. Min. Proc. Inst. Civil Eng. 30, 28.

[4] Douglass JN 1871 On the wolf rock lighthouse. Min. Proc. Inst. Civil Eng. 49, 214–227.

[5] Boyle M 1997 Wolf rock, 1st edn Southampton, UK: B&T Publications.

[6] Watson G 1988 The civils, p. 251 London, UK: Thomas Telford Ltd.

[7] Nicholson C 2015 Rock lighthouses of Britain. Dunbeath, UK: Whittles Publishing.

[8] Smeaton J 1791 *A narrative of the building and a description of the construction of the Eddystone lighthouse with stone, published by John Smeaton*.

[9] Grant HK 1969 Robert Stevenson, engineer and sea builder. New York, NY: Meredith Press.

[10] Morison JR, O'Brien MP, Johnson JW, Schaaf SA 1950 The force exerted by surface waves on piles. Petrol. Trans. Am. Inst. Min. Eng. 189, 149–154. (10.2118/950149-G)

[11] Trinity House. 1973 Court minutes, pp. 184. CLC/526/MS300004/042.

[12] Ewins DJ 2000 Modal testing: theory, practice and application. Baldock, Hertfordshire, UK: Research Studies Press Ltd.

[13] Richardson MH, Formenti DL 1985 Global curve fitting of frequency response measurements using the rational fraction polynomial method. In IMAC III, Orlando, FL, USA, pp. 390–397.

[14] Kennedy CC, Pancu CDP 1947 Use of vectors in vibration measurement and analysis. J. Aeronaut. Sci. 14, 603–625. (10.2514/8.1474)

[15] Au SK, Zhang F-L, Ni Y-C 2013 Bayesian operational modal analysis: theory, computation, practice. Comput. Struct. 126, 3–14. (10.1016/j.compstruc.2012.12.015)

[16] den Hartog JP 1985 Mechanical vibrations. Mineola, NY: Dover Publications Inc.

[17] Brownjohn JMW, Raby A, Bassitt J, Antonini A, Hudson E, Dobson P 2018 Experimental modal analysis of British rock lighthouses. Mar. Struct. 62, 1–22. (10.1016/j.marstruc.2018.07.001)

[18] Fawcett L, Walshaw D 2012 Estimating return levels from serially dependent extremes. Environmetrics 23, 272–283. (10.1002/env.2133)

[19] Sartini L, Mentaschi L, Besio G 2015 Comparing different extreme wave analysis models for wave climate assessment along the Italian coast. Coast. Eng. 100, 37–47. (10.1016/j.coastaleng.2015.03.006)

[20] Teena NV, Sanil Kumar V, Sudheesh K, Sajeev R 2012 Statistical analysis on extreme wave height. Nat. Hazards 64, 223–236. (10.1007/s11069-012-0229-y)

[21] Silva GAM, Mendes D 2013 Comparison results for the cfsv2 hindcasts and statistical downscaling over the northeast of Brazil. Adv. Geosci. 35, 79–88. (10.5194/adgeo-35-79-2013)

[22] Antonini A, Archetti R, Lamberti A 2017 Wave simulation for the design of an innovative quay wall: the case of Vlorë harbour. Nat. Hazards Earth Syst. Sci. 17, 127–142. (10.5194/nhess-17-127-2017)

[23] Boudière E et al. 2013 A suitable metocean hindcast database for the design of marine energy converters. Int. J. Marine Energy 3–4, e40–e52. (10.1016/j.ijome.2013.11.010)

[24] Antonini A, Raby A, Brownjohn JMW, Pappas A, D'Ayala D 2019 Survivability assessment of Fastnet lighthouse. Coast. Eng. 150, 18–38. (10.1016/j.coastaleng.2019.03.007)

[25] Bricheno LM, Wolf J 2018 Future wave conditions of Europe, in response to high-end climate change scenarios. J. Geophys. Res.: Oceans 123, 8762–8791. (10.1029/2018JC013866)

[26] Aarnes OJ et al. 2017 Projected changes in significant wave height toward the end of the 21st century: Northeast Atlantic. J. Geophys. Res. Oceans 122, 3394–3403. (10.1002/2016JC012521)

[27] Pickands J 1975 Statistical inference using extreme order statistics. Ann. Stat. 3, 119–131. (10.1214/aos/1176343003)

[28] Méndez FJ, Menéndez M, Luceño A, Losada IJ 2006 Estimation of the long-term variability of extreme significant wave height using a time-dependent peak over threshold (POT) model. J. Geophys. Res. 11, C07024 (10.1029/2005JC003344)

[29] Cunnane C 1979 A note on the Poisson assumption in partial duration series models. Water Resour. Res. 15, 489–494. (10.1029/WR015i002p00489)

[30] Ferro CAT, Segers J 2003 Inference for clusters of extreme values. J. R. Stat. Soc. Series B: Stat. Methodol. 65, 545–556. (10.1111/1467-9868.00401)

[31] Cheng L, AghaKouchak A, Gilleland E, Katz RW 2014 Non-stationary extreme value analysis in a changing climate. Clim. Change 127, 353–369. (10.1007/s10584-014-1254-5)

[32] Bromirski PD, Cayan DR 2015 Wave power variability and trends across the North Atlantic influenced by decadal climate patterns. J. Geophys. Res.: *Oceans* 120, 3419–3443. (10.1002/2014JC010440)

[33] Wang XL, Swail VR 2001 Changes of extreme wave heights in Northern Hemisphere oceans and related atmospheric circulation regimes. J. Clim. 14, 2204–2221. (10.1175/1520-0442(2001)014<2204:COEWHI>2.0.CO;2)

[34] Gilleland E, Katz RW 2016 ExtRemes 2.0: an extreme value analysis package. J. Stat. Softw. 72, 1–39. (10.18637/jss.v072.i08)

[35] Santo H, Taylor PH, Gibson R 2016 Decadal variability of extreme wave height representing storm severity in the northeast Atlantic and North Sea since the foundation of the Royal Society. Proc. R. Soc. A 472, 20160376 (10.1098/rspa.2016.0376) 27713662 PMC5046986

[36] Stephenson A, Tawn J 2004 Bayesian inference for extremes: accounting for the three extremal types. Extremes 7, 291–307. (10.1007/s10687-004-3479-6)

[37] Braak CJFT 2006 A Markov chain Monte Carlo version of the genetic algorithm differential evolution: easy Bayesian computing for real parameter spaces. Stat. Comput. 16, 239–249. (10.1007/s11222-006-8769)

[38] Gelman A, Shirley K 2011 Inference from simulations and monitoring convergence. In Handbook of Markov chain Monte Carlo. London, UK: Chapman & Hall/CRC.

[39] Scotto MG, Guedes SC 2007 Bayesian inference for long-term prediction of significant wave height. Coast. Eng. 54, 393–400. (10.1016/j.coastaleng.2006.11.003)

[40] Gibson R 2011 A hierarchical Bayesian spatial directional model for wave heights and structural response. In Proc. of the 12th Int. Workshop on Wave Hindcasting and Forecasting HI: World Meteorological Organization, *Kona, Hawaii, 30 October–4 November 2011*. Geneva: World Meteorological Organization.

[41] Viselli AM, Forristall GZ, Pearce BR, Dagher HJ 2015 Estimation of extreme wave and wind design parameters for offshore wind turbines in the Gulf of Maine using a POT method. Ocean Eng. 104, 649–658. (10.1016/j.oceaneng.2015.04.086)

[42] Goda Y 2000 Random seas and design of maritime structures, 2nd edn Singapore, Singapore: World Scientific Publishing Co.

[43] Battjes JA, Groenendijk HW 2000 Wave height distributions on shallow foreshores. Coast. Eng. 40, 161–182. (10.1016/S0378-3839(00)00007-7)

[44] Miche R 1944 Mouvements Ondulatoires des Mers en Profondeur Constante et Decroisante. Ann. des Ponts et Chaussees 114, 25–78, 131–164, 270–292, 369–406.

[45] Munk WH 1949 The solitary wave theory and its application to surf problems. Ann. NY Acad. Sci. 5, 376–424. (10.1111/j.1749-6632.1949.tb27281.x)

[46] Goda Y 1970 Numerical experiments on wave statistics with spectral, vol. 9 Yokosuka, Japan: Port and Harbour Research Institute.

[47] CERC. 1984 Shore protection manual. Vicksburg, MS: Coastal Engineering Research Center, US Corps of Engineer.

[48] Palmer M et al. 2018 UKCP18 marine report. Exeter, UK: Met Office See https://ukclimateprojections.metoffice.gov.uk.

[49] Hansen JB 1990 Periodic waves in the surf zone: analysis of experimental data. Coast. Eng. 14, 19–41. (10.1016/0378-3839(90)90008-K)

[50] Wienke J, Oumeraci H 2005 Breaking wave impact force on a vertical and inclined slender pile—theoretical and large-scale model investigations. Coast. Eng. 52, 435–462. (10.1016/j.coastaleng.2004.12.008)

[51] Grimshaw RHJ 1971 The solitary wave in water of variable depth, part 2. J. Fluid Mech. 46, 611–622. (10.1017/S0022112071000739)

[52] Pappas A, D'Ayala D, Antonini A, Raby A 2018 Rock mounted iconic lighthouses under extreme wave impacts: limit analysis and discrete element method. In 9th Int. Conf. Computational Methods, Rome.

[53] Pappas A, D'Ayala D, Antonini A, Raby A 2018 Finite element modelling and limit analysis of Fastnet lighthouse under impulsive ocean waves. In Structural Analysis of Historical Constructions, *RILEM Bookseries* (eds R Aguilar, D Torrealva, S Moreira, MA Pando, LF Ramos), vol. 18, pp. 881–890. Basel, Switzerland: Springer International Publishing.

[54] Abaqus. 2014 Abaqus documentation—version 6.14. Providence, RI: Dassault Systèmes.

[55] Trinity House. 1869 *Wolf rock construction drawings*, n.3323–n.3363.

[56] Trinity House. 1973 *Wolf rock helideck drawings*, n.85/131–n.85/139.

[57] Pappas A et al 2017 Numerical modelling of Fastnet lighthouse based on experimental dynamic identification. In Int. Conf. on Advances in Construction Materials and Systems, Chennai.

[58] British Standards Institution. 1990 *BS 4360:1990 Weldable structural steels*.

[59] EN 1993-1-1. 2007 *UNI EN 1993-1-1: Eurocode 3: design of steel structures—Part 1-1: general rules and rules for buildings*. 44.

[60] Itasca Inc. 2013 *3DEC 5.0: 3-dimensional distinct element code, theory and background*.

[61] Banfi D, Raby A, Simmonds D 2019 Dynamic loads arising from broken wave impacts on a cylindrical turbine substructure in shallow waters. In 13th EWTEC, Napoli.

